# Nanostructured 3D‐Printed Hybrid Scaffold Accelerates Bone Regeneration by Photointegrating Nanohydroxyapatite

**DOI:** 10.1002/advs.202300038

**Published:** 2023-03-11

**Authors:** Lei Tong, Xiaocong Pu, Quanying Liu, Xing Li, Manyu Chen, Peilei Wang, Yaping Zou, Gonggong Lu, Jie Liang, Yujiang Fan, Xingdong Zhang, Yong Sun

**Affiliations:** ^1^ National Engineering Research Center for Biomaterials Sichuan University 29^#^ Wangjiang Road Chengdu 610064 China; ^2^ Sichuan Testing Center of Medical Devices Sichuan Institute for Drug Control NMPA Key Laboratory for Technical Research on Drug Products In Vitro and In Vivo Correlation 8# Xinwen Road Chengdu 611731 China; ^3^ Department of Neurosurgery West China Hospital Sichuan University 37# Guoxue Lane Chengdu 610041 China; ^4^ Sichuan Testing Center for Biomaterials and Medical Devices Sichuan University 29# Wangjiang Road Chengdu 610064 China

**Keywords:** 3D printing, bone regeneration, hydrogels, nanohydroxyapatite, photoinitiation

## Abstract

Nanostructured biomaterials that replicate natural bone architecture are expected to facilitate bone regeneration. Here, nanohydroxyapatite (nHAp) with vinyl surface modification is acquired by silicon‐based coupling agent and photointegrated with methacrylic anhydride‐modified gelatin to manufacture a chemically integrated 3D‐printed hybrid bone scaffold (75.6 wt% solid content). This nanostructured procedure significantly increases its storage modulus by 19.43‐fold (79.2 kPa) to construct a more stable mechanical structure. Furthermore, biofunctional hydrogel with biomimetic extracellular matrix is anchored onto the filament of 3D‐printed hybrid scaffold (HGel‐*g*‐nHAp) by polyphenol‐mediated multiple chemical reactions, which contributes to initiate early osteogenesis and angiogenesis by recruiting endogenous stem cells in situ. Significant ectopic mineral deposition is also observed in subcutaneously implanted nude mice with storage modulus enhancement of 25.3‐fold after 30 days. Meanwhile, HGel‐*g*‐nHAp realizes substantial bone reconstruction in the rabbit cranial defect model, achieving 61.3% breaking load strength and 73.1% bone volume fractions in comparison to natural cranium 15 weeks after implantation. This optical integration strategy of vinyl modified nHAp provides a prospective structural design for regenerative 3D‐printed bone scaffold.

## Graphical abstract

The photointegration of vinyl‐modified nanohydroxyapatite and gelatin significantly increases the storage modulus of 3D‐printed scaffold in comparison with traditional hydroxyapatite as bioink, and the anchored biomimetic extracellular matrix on the surface of filament based on polyphenol‐mediated multiple reactions synergistically enhances the substantial bone regeneration by availably initiating endogenous stem cell recruitment and osteogenic/angiogenic differentiation.

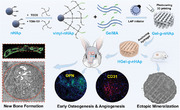

## 1 Introduction

Inevitable limitations of autologous and allogeneic bone grafts necessitate the development of functional artificial bony implants with biomimetic composition and structure.^[^ ^1^, ^2^ ^]^ Therein, exogenous cells and growth factors in bone tissue engineering (BTE) scaffolds present an unpredictable clinical risk, constraining their applications in bone regeneration.^[^ ^3^ ^]^ Many cell/factor‐free scaffolds with optimized material designs have made progress by spontaneously recruit endogenous stem cells (ESCs) in situ to guide rapid vascularization and early osteogenesis,^[^ ^4^, ^5^, ^6^ ^]^ such as integrating bioactive hydrogels mimicking a natural extracellular matrix (ECM) in hydroxyapatite composite scaffolds.^[^ ^7^, ^8^ ^]^ However, the hydroxyapatite composite scaffold's weak interfacial integration and structural instability diminish bone repair.^[^ ^9^ ^]^ Therefore, improving the microstructural design of hydroxyapatite composite scaffold is expected to enhance bone healing efficacy.

Within natural bone's mineralized collagen fiber nanostructure, nanohydroxyapatite (nHAp) crystals orderly form rigid bones by embedding the gaps (40 nm) between collagen molecules.^[^ ^10^, ^11^ ^]^ Inspired by this, a simulated structure of nanoscale mineralized collagen fiber is instructive for designing BTE scaffolds.^[^ ^12^, ^13^, ^14^ ^]^ Currently, nanostructured scaffolds manufactured from nanoparticles,^[^ ^15^ ^]^ nanofibers,^[^ ^16^ ^]^ nanotubes,^[^ ^17^ ^]^ and other nanomaterials have emerged as promising candidates for improving conventional BTE scaffolds.^[^ ^18^ ^]^ Among them, the development of 3D printing (3DP) technology provides an advanced choice for constructing nanofiber structured BTE scaffold, which realizes small‐scale sophisticated biomanufacturing to replicate natural bone structure.^[^ ^19^, ^20^ ^]^ However, the particle size of hydroxyapatite in composite fibers is generally micron scale in many cases, and it lacks effective integration with the composite matrix, which deviates substantially from the nanoscale organic/inorganic integration in the natural bone tissue and affects its long‐term biological function.^[^ ^21^, ^22^ ^]^ Therefore, integrating nanosized hydroxyapatite into 3DP fiber matrix has the potential to enhance the osteogenesis of 3DP BTE scaffolds.

Methacrylic anhydride‐modified gelatin (GelMA), a partial collagen hydrolysis product, provides substantial advantages to BTE scaffolds due to its superior biocompatibility, desirable cell adhesion ability, photoprintability, and biodegradability.^[^ ^23^, ^24^ ^]^ Combining GelMA with nHAp is a promising 3DP material design strategy.^[^ ^25^ ^]^ However, undesirable dispersion of nHAp and its weak interfacial interactions with composite matrix result in inefficient biomineralization and dissatisfied osseointegration.^[^ ^26^ ^]^ Various strategies, such as biomimetic mineralization,^[^ ^27^, ^28^ ^]^ organic–inorganic hybridization,^[^ ^29^ ^]^ and particle size adjustment of hydroxyapatite^[^ ^30^ ^]^ have been explored to enhance the interfacial interactions between the composite matrix and nHAp. Free‐radical photoinitiated polymerization has been widely used to prepare shape‐tailored scaffolds because of its benefits of improved structuring and enhanced mechanical properties.^[^ ^31^ ^]^ Hence, incorporating nHAp with surface modification into photoinitiated systems to improve its interfacial interaction with GelMA is an effective approach to enhance osseointegration and bone regeneration.^[^ ^32^ ^]^ Silane coupling agents have been widely reported for surface modification of inorganic nanoparticles (e.g., bioactive glass,^[^ ^33^ ^]^ hydroxyapatite^[^ ^34^, ^35^, ^36^ ^]^), which could adjust their surface structure and enhance their dispersion in composite matrix by imparting functional groups.^[^ ^37^ ^]^ Besides, silicon as an essential trace element participates in bone tissue regeneration.^[^ ^38^ ^]^ Some studies have shown that silicon‐enhanced biomaterials promoted bone healing by guiding bone marrow stem cells (BMSCs) differentiation, activating intercellular interactions, and stimulating angiogenesis.^[^ ^39^, ^40^, ^41^ ^]^ Therefore, we expected that the introduction of silane coupling agent could confer functional groups on nHAp surface to enhance interfacial integration with 3DP composite matrix and synchronously improve osteogenic efficacy.

**Scheme 1:**
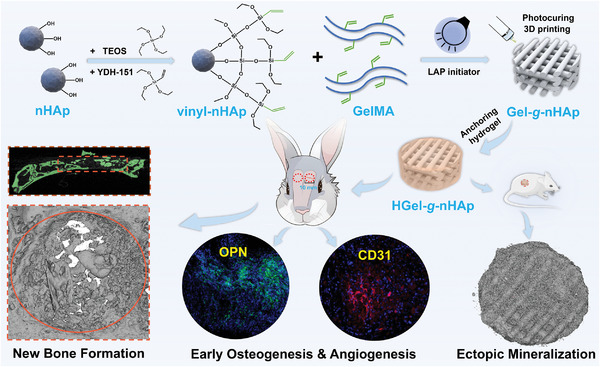
Schematic illustration of nanostructured 3DP hybrid scaffold for bone regeneration.

Here, nHAp was surface modified by vinyl‐based silane coupling agent, and coupled with GelMA via double bonding under photoinitiation to prepare a chemically integrated gelatin‐grafted nHAp 3DP scaffold (Gel‐*g*‐nHAp) that provides mechanical support and shape matching for BTE scaffolds. Furthermore, dopamine‐mediated functional hydrogel mimicking ECM was anchored to Gel‐*g*‐nHAp scaffolds to create mechanical and biofunctional bone implant (HGel‐*g*‐nHAp) with multireactive polyphenols bound to amino groups of GelMA and collagen via Michael addition reactions and to hydroxyapatite via Ca^2+^ chelation acting as bridges between organic–inorganic components.^[^ ^6^ ^]^ Nanostructured procedure was expected to provide better mechanical support and biological regulatory functions (**Scheme** **1**). In vitro cellular assays and nude mouse subcutaneous ectopic osteogenesis were employed to analyze BMSCs proliferation and differentiation, as well as mineralized depositional capacity. A rabbit cranial defect model was also used to confirm nanostructured scaffold‐induced bone regeneration.

## 2 Results

### 2.1 Characterization of Vinyl‐Modified nHAp

**Figure 1:**
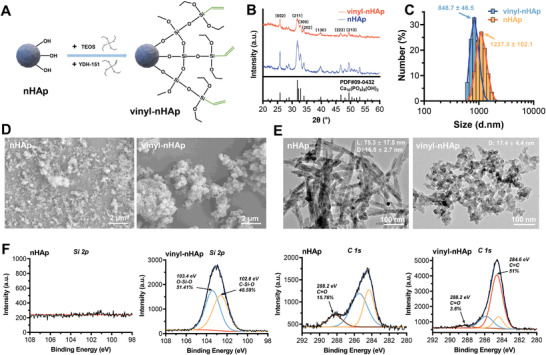
Successful synthesis of vinyl‐modified nHAp. A) Schematic of the synthesis of vinyl‐nHAp. B) X‐ray diffraction spectra of nHAp/vinyl‐nHAp and standard spectra of Ca_10_(PO_4_)_6_(OH)_2_. C) Size distribution of nHAp/vinyl‐nHAp measured by dynamic light scattering (*n* = 3). D) Representative SEM images and E) TEM images of nHAp/vinyl‐nHAp. F) High‐resolution X‐ray photoelectron spectroscopy spectra of Si 2p and C 1s in nHAp/vinyl‐nHAp.

Vinyl‐modified nanohydroxyapatite (vinyl‐nHAp) was prepared by grafting tetraethyl orthosilicate (TEOS) and triethoxyvinylsilane (silane coupling agent YDH‐151) onto the nHAp surface (**Figure** **1**A). A series of vinyl‐nHAp was prepared in solutions with ethanol:water ratios of 6:1, 7:1, 8:1, and 9:1. Vinyl‐nHAp's new C=C stretching vibrational peak (1610 cm^−1^) and C—H bending vibrational peak (1416, 1276, and 768 cm^−1^) in the Fourier‐transform infrared (FT‐IR) spectroscopy spectra (Figure S1, Supporting Information) confirmed that the nHAp had been successfully grafted with vinyl. An iodine titration method was used to quantify the vinyl content of vinyl‐nHAp (Figure S2A, Supporting Information), which increased significantly with increasing ethanol:water ratio in the reaction solution, with the highest vinyl content of 8.3 × 10^−3^ mol g^−1^ in the 9:1 group. During thermal weight loss from room temperature to 800 °C (Figure S2B, Supporting Information), vinyl‐nHAp exhibited lower mass maintenance than nHAp, with a minimum of 89.7% in the 9:1 group and a 5.4% excess weight loss in comparison with nHAp, reflecting the silane coupling agent and vinyl groups grafted onto the nHAp surface. These results indicated that the 9:1 group had the highest vinyl grafting rate, which was attributed to the increased ethanol content favoring the hydrolysis of TEOS and YDH‐151, resulting in a more complete condensation reaction with the surface hydroxyl groups of nHAp. Therefore, vinyl‐nHAp (9:1) with the highest vinyl content was used for subsequent experiments.

X‐ray diffraction (XRD, Figure 1B) showed that vinyl‐nHAp still had strong diffraction peak intensities in (211), (002), and (300) crystal faces, which was consistent with hydroxyapatite's standard card (PDF#09‐0432), indicating that the modification did not destroy its physical phase structure. However, the intensity of all diffraction peaks decreased after the modification, indicating a decrease in its crystallinity. The particle size quantified by dynamic light scattering (DLS; Figure 1C) revealed that the vinyl modification decreased nHAp size and agglomeration. HAp's morphology before and after modification was also determined using scanning electron microscopy (SEM), and transmission electron microscopy (TEM), as shown in Figure 1D,E. The needle‐shaped structure of nHAp was converted to an irregular spherical structure with an average granularity of 17.4 ± 4.4 nm (Figure S3, Supporting Information) after modification. The nHAp with a needle‐shaped crystal structure might exhibit higher frictional resistance as 3DP ink, while vinyl‐nHAp with irregular spherical structure might be more suitable for printing ink extrusion. X‐ray photoelectron spectroscopy (XPS) was used to verify the molecular structure of vinyl‐nHAp (Figure 1F). Compared with nHAp, newly appearing Si 2p peaks (O—Si—O, 103.4 eV, 51.41% and C—Si—O, 102.6 eV, 48.59%) and higher C 1s peaks (newly appearing C=C, 284.6 eV, 51%) were observed in vinyl‐nHAp, confirming the substantial presence of silicon element and vinyl.

### 2.2 Characterization of Nanostructured 3DP Scaffolds

Bioinks for 3DP were prepared by mixing GelMA, hydroxyapatite, and photoinitiator lithium phenyl (2,4,6‐trimethylbenzoyl) phosphinate (LAP). At a sufficiently high hydroxyapatite content (75.6 wt%) to provide sufficient osteoinductive ability, the bioink composed of GelMA and nHAp slurry without surface modification (Gel‐nHAp) was difficult to extrude for printing due to its viscosity, yet the Gel‐*g*‐nHAp bioink still had an appropriate viscosity and maintained good printability and customizability (Figure S4, Supporting Information). SEM images (Figure S5, Supporting Information) showed that GelMA had microsized lamellar structures. In contrast, vinyl‐nHAp in Gel‐*g*‐nHAp bioink was uniformly distributed on GelMA and formed a dense, crosslinked structure through potential chemical bonding.

**Figure 2:**
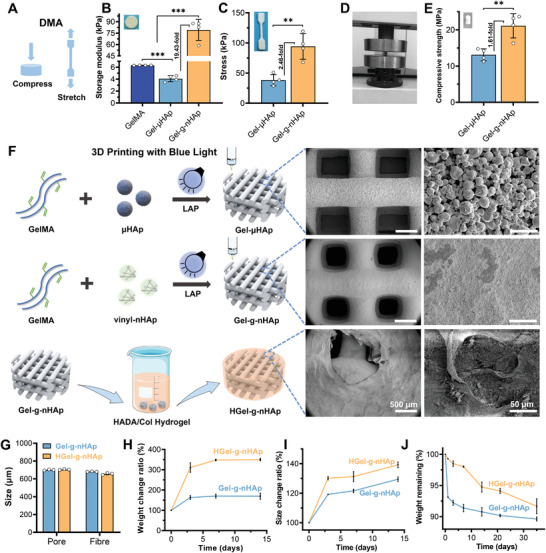
Preparation and characterization of nanostructured 3DP hybrid scaffolds. A) Schematic diagram of compressive elasticity and tensile strength of bioinks tested by dynamic mechanical analysis (*n* = 4). B) Compressive storage modulus and C) tensile strength of different bioinks. D) Universal testing machine for compressive strength testing (*n* = 4). E) Compressive strength of different bioinks. F) Manufacturing schematic of 3D‐printed Gel‐µHAp, Gel‐*g*‐nHAp, and HGel‐*g*‐nHAp and their micromorphology observed by SEM. G) Pore size and fiber size of Gel‐*g*‐nHAp and HGel‐*g*‐nHAp scaffolds (*n* = 3). H,I) Quantitative analysis (*n* = 3) of swelling test in PBS. J) Degradation curves in Tris‐HCl. Significance level was set as *p* < 0.05 (*), *p* < 0.01 (**), and *p* < 0.001 (***).

Different bioinks were made into circular and dumbbell‐shaped samples to test their compressive elastic modulus and tensile strength (**Figure** **2**A). Elasticity was evaluated by measuring its storage modulus using compression mode of dynamic mechanical analysis (DMA). The storage modulus of compared Gel‐µHAp (preparation by physical blending GelMA and micron grade hydroxyapatite) was lower than GelMA (Figure 2B), indicating that adding hydroxyapatite reduced the elasticity of bioink, opposite to the solid content enhancement. Gel‐*g*‐nHAp exhibited a highest elastic modulus of 79.2 ± 13.9 kPa since vinyl‐nHAp ensured the solid content and elasticity of bioinks by forming nanoscale covalent crosslinks with GelMA. Tensile tests (Figure S6A, Supporting Information) also showed a similar trend: Gel‐*g*‐nHAp had better tensile properties than Gel‐µHAp. At a specific strain of 9.8% (Figure 2C), Gel‐*g*‐nHAp had a significantly higher tensile strength (94.2 ± 18.4 kPa) than Gel‐µHAp (38.3 ± 8.1 kPa). A universal testing machine was used to test the compressive strength (Figure 2D). The results (Figure S6B, Supporting Information and Figure 2E) showed that Gel‐*g*‐nHAp had a significantly higher compressive strength. These above results could be attributed to the double bonding between vinyl‐nHAp and GelMA formed by photocrosslinking, allowing hydroxyapatite to be more uniformly distributed in GelMA.

As shown in Figure 2F, the bioink composed of GelMA, vinyl‐nHAp, and LAP constructed a nanostructured organic–inorganic hybrid scaffold with chemically covalent structures by simple photoinitiated direct ink writing, and fabricated scaffolds with 0°/90° alternating angle pattern structures. In gross view and SEM images of 3DP scaffolds, Gel‐*g*‐nHAp showed a denser surface than Gel‐µHAp. Furthermore, catechol‐mediated polysaccharide hydrogel matrix composed of dopamine‐modified hyaluronic acid (HADA) and Collagen type I (Col I) was anchored to Gel‐*g*‐nHAp by chemical crosslinking to form a hydrogel anchored scaffold (HGel‐*g*‐nHAp) for enhancing its mechanical properties and biological functions. The surface of HGel‐*g*‐nHAp was covered by a porous hydrogel layer (Figure S7, Supporting Information). The pore structure parameters (Figure 2G) did not differ significantly between groups, around 700 µm. Swelling experiments were performed in phosphate‐buffered saline (PBS) solution (Figure 2H,I). The size and weight ratio changes were significantly greater with HGel‐*g*‐nHAp than Gel‐*g*‐nHAp scaffolds, attributed to the water storage capacity of porous hydrogel layer. In degradation experiments performed in PBS (Figure 2J), the HGel‐*g*‐nHAp group maintained weight stability during the early degradation stage. These results suggested that the hydrogel layer delays surrounding media invasion and prevents excessive scaffold degradation during the early implantation stage through its water storage capacity.

### 2.3 HGel‐*g*‐nHAp Promotes BMSCs Proliferation and Osteogenic Differentiation In Vitro

**Figure 3:**
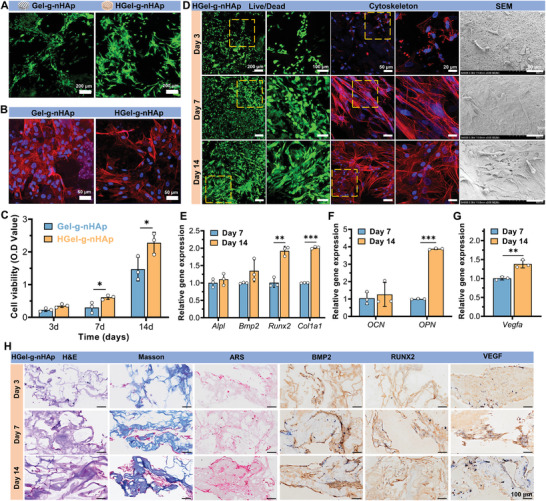
BMSCs proliferation and osteogenic differentiation in vitro. A) Live/dead staining and B) cytoskeleton staining of BMSCs‐laden Gel‐*g*‐nHAp and HGel‐*g*‐nHAp scaffolds at 5 days. C) BMSCs viability of Gel‐*g*‐nHAp and HGel‐*g*‐nHAp scaffolds at 3, 7, and 14 days measured by CCK‐8 (*n* = 3). D) Live/dead staining, cytoskeleton staining, and SEM images of BMSCs on HGel‐*g*‐nHAp scaffolds at 3, 7, and 14 days. E) Relative expression of *Alpl*, *Bmp2*, *Runx2*, and *Col1a1* showing early osteogenesis level. F) Relative expression of *OCN* and *OPN* showing late osteogenesis level. G) Relative expression of *Vegfa* showing angiogenesis level. H) Representative H&E, Masson's trichrome, ARS, and IHC (BMP2, RUNX2, and VEGF) staining evaluating osteogenic and angiogenic differentiation of BMSCs on HGel‐*g*‐nHAp scaffolds. Scale bar: 100 µm. Significance level was set as *p* < 0.05 (*), *p* < 0.01 (**), and *p* < 0.001 (***).

Gel‐*g*‐nHAp and HGel‐*g*‐nHAp scaffolds cocultured with BMSCs for 5 days were subjected to live/dead staining (**Figure** **3**A) and cytoskeleton staining (Figure 3B) to assess the introduced bioactive hydrogel's effect on osteogenic activity. Distinct orientation growth was observed on the Gel‐*g*‐nHAp scaffold due to restricted growth areas. BMSCs on the HGel‐*g*‐nHAp scaffold showed greater proliferation and adhesion since the introduced bioactive hydrogel matrix provided a superior microenvironment for cell growth. The Cell Counting Kit‐8 results (Figure 3C) also showed that BMSCs showed greater proliferation on the HGel‐*g*‐nHAp after 7 days. Furthermore, HGel‐*g*‐nHAp was subjected to live/dead, cytoskeleton staining, and SEM observing after coculturing with BMSCs for 3, 7, and 14 days. According to Figure 3D, the live/dead cell staining showed almost no visible dead cells and progressive live cell proliferation, indicating HGel‐*g*‐nHAp has a desirable biocompatibility. In addition, the spreading area of BMSCs gradually increased, suggesting that HGel‐*g*‐nHAp promoted BMSCs proliferation, adhesion, and spreading.

The expression levels of genes related to early‐stage osteogenesis (biomineralization associated alkaline phosphatase [*Alpl*], bone morphogenetic protein 2 [*Bmp2*], RUNX family transcription factor 2 [*Runx2*], and collagen type I alpha 1 [*Col1a1*]), angiogenesis (vascular endothelial growth factor A [*Vegfa*]), and late‐stage osteogenesis (bone gamma‐carboxyglutamate protein [*Bglap*/*OCN*] and secreted phosphoprotein 1 [*Spp1*/*OPN*]) were measured by quantitative polymerase chain reaction (qPCR) to assess BMSCs osteogenic and angiogenic differentiation after 7 and 14 days of in vitro coculturing (Figure 3E–G). The expression of all genes gradually increased up to day 14, especially *Runx2*, *Col1a1*, *Vegfa*, and *OPN*, which were significantly higher on day 14 than on day 7.

To further observe the osteogenic differentiation, HGel‐*g*‐nHAp scaffolds cocultured with BMSCs for 3, 7, and 14 days were sectioned and stained using hematoxylin and eosin (H&E), Masson's trichrome, and Alizarin Red S (ARS), and subjected to immunohistochemistry (IHC). Gradual increment of cell numbers (H&E), mature collagen matrix secretion (Masson), and calcium nodule formation (ARS) were observed with increasing coculturing time. In addition, BMP2, RUNX2, and VEGF expression gradually increased with visual improvements in osteogenesis and angiogenesis (Figure 3H). These results showed that the HGel‐*g*‐nHAp scaffold could promote BMSCs proliferation and osteogenic differentiation in vitro.

### 2.4 HGel‐*g*‐nHAp Promotes Early Osteogenesis by Recruiting Endogenous Stem Cells

**Figure 4:**
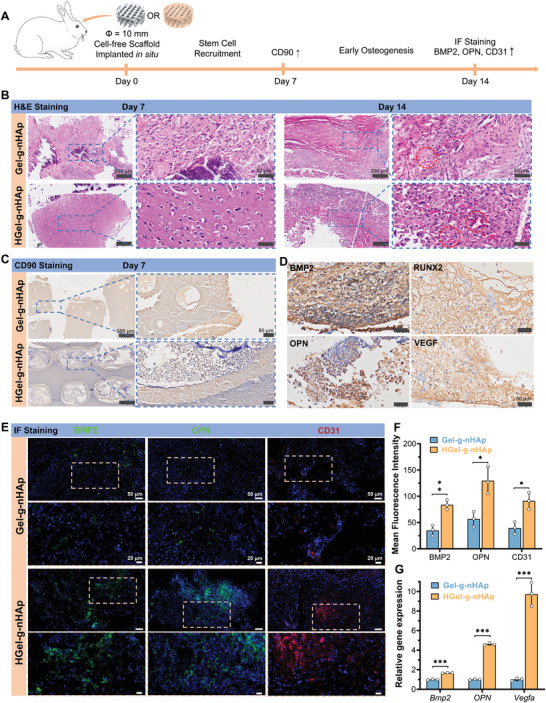
Endogenous stem cell recruitment and early osteogenesis in vivo. A) Schematic diagram of cell recruitment experiments (*n* = 3). B) H&E staining of Gel‐*g*‐nHAp and HGel‐*g*‐nHAp scaffolds after 7 and 14 days of implantation. Scale bar: 200, 50 µm. C) IHC staining of CD90 at day 7 showing more stem cells infiltration in HGel‐*g*‐nHAp scaffolds. Scale bar: 500, 50 µm. D) IHC staining of BMP2, Runx2, OPN, and VEGF in HGel‐*g*‐nHAp scaffolds after 7 days of implantation. Scale bar: 50 µm. E) IF staining of BMP2, OPN, and CD31 at day 14 showing higher early osteogenesis and angiogenesis in HGel‐*g*‐nHAp scaffolds. Scale bar: 50, 20 µm. F) Semiquantitative results of IF staining. G) Relative expression of *Bmp2, OPN*, and *Vegfa* at day 14. Significance level was set as *p* < 0.05 (*), *p* < 0.01 (**), and *p* < 0.001 (***).

Cell‐free Gel‐*g*‐nHAp and HGel‐*g*‐nHAp scaffolds were implanted into rabbit cranial critical‐size defects (Φ = 10 mm) and taken out for assessment on days 7 and 14 (**Figure** **4**A). H&E staining (Figure 4B) showed that more endogenous cells had been recruited on day 14 in comparison with day 7 in both groups. In addition, better cellular infiltration and neovascularization (red circle) were observed in the HGel‐*g*‐nHAp group, indicating that HGel‐*g*‐nHAp provided a better microenvironment for cellular infiltration and angiogenesis. IHC staining for cluster of differentiation 90 (CD90; a stem cell surface marker) on day 7 showed higher positive expression in the HGel‐*g*‐nHAp than the Gel‐*g*‐nHAp group (Figure 4C). This result highlighted the significance of the bioactive hydrogel matrix in recruiting ESCs in vivo. Furthermore, the HGel‐*g*‐nHAp group was subjected to IHC staining for BMP2, RUNX2, OPN, and VEGF (Figure 4D). All showed varying degrees of positive expression, indicating that the recruited ESCs exhibited osteogenesis and angiogenesis potential at an early stage.

Osteogenic potential of scaffolds was further evaluated by immunofluorescence staining on day 14. The HGel‐*g*‐nHAp group showed greater BMP2, OPN, and CD31 fluorescence than the Gel‐*g*‐nHAp group (Figure 4E). A semiquantitative Image J analysis of mean fluorescence intensity (Figure 4F) indicated that HGel‐*g*‐nHAp had higher osteogenic and angiogenic potential than Gel‐*g*‐nHAp. Furthermore, the qPCR results (Figure 4G) showed that expression of osteogenesis‐related (*Bmp2* and *OPN*) and angiogenesis‐related (*Vegfa*) genes were significantly higher in the HGel‐*g*‐nHAp group than in the Gel‐*g*‐nHAp group. These results suggested that the introduction of bioactive hydrogel matrix into the 3DP Gel‐*g*‐nHAp scaffold could stimulate rapid vascularization and early osteogenesis by recruiting ESCs in situ.

### 2.5 HGel‐*g*‐nHAp Promotes Ectopic Osteogenesis In Vivo

**Figure 5:**
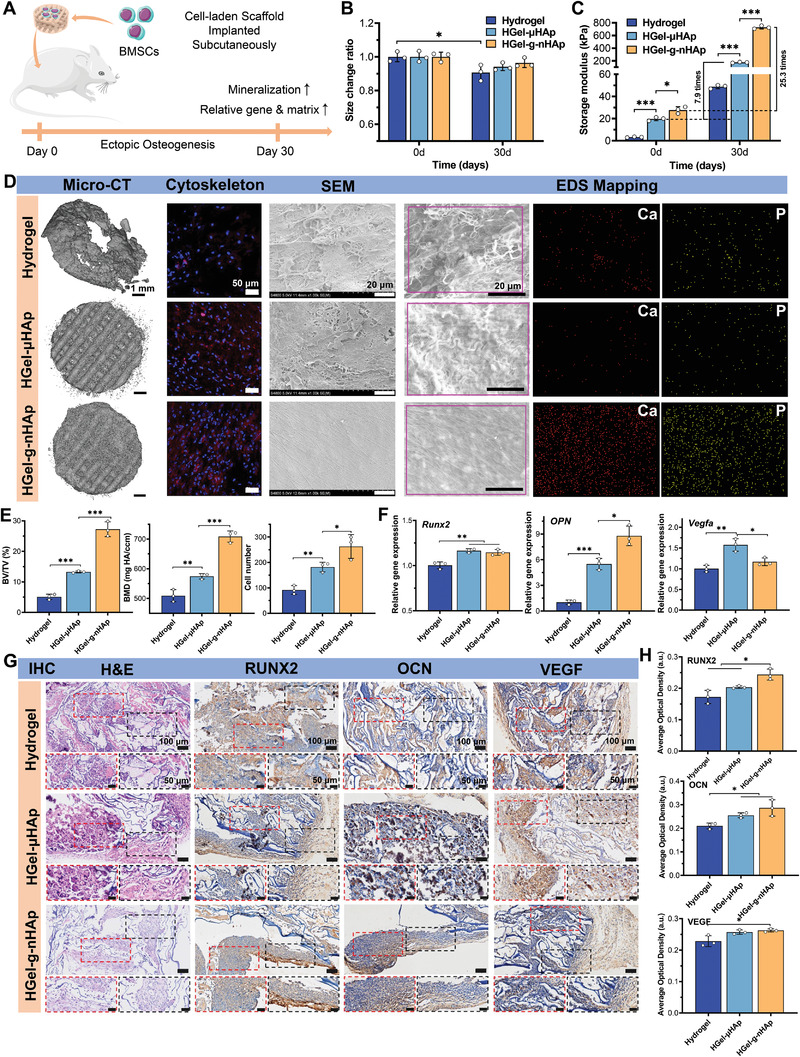
Ectopic osteogenesis of different scaffolds in subcutaneous implantation model. A) Schematic diagram of ectopic osteogenesis experiments (*n* = 3). B) Dimensional statistics of different scaffolds before and after implantation. C) Compressive storage modulus of different scaffolds indicating mechanical enhancements. D) Micro‐CT 3D reconstruction, cytoskeleton images, SEM, and EDS mapping images showing ectopic osteogenesis of different scaffolds. E) Semiquantitative results of bone volume fraction (BV/TV), bone mineral density (BMD), and cell number. F) Relative expression of *Runx2, OPN*, and *Vegfa*. G) Representative H&E, IHC staining in different scaffolds after implantation. Scale bar: 100, 50 µm. H) Semiquantitative results of IHC staining. Significance level was set as *p* < 0.05 (*), *p* < 0.01 (**), and *p* < 0.001 (***).

To assess the mineral deposition and ectopic osteogenesis capacities of HGel‐*g*‐nHAp, three groups of scaffolds were subcutaneously implanted into BALB/c nude mice for 30 days (**Figure** **5**A). HGel‐µHAp and HADA/Col hydrogel were used as control groups. All scaffolds were partially fused with the surrounding tissue on day 30. Statistics for the sizes before and after implantation are shown in Figure 5B. The hydrogel group showed significant dimensional reduction after implantation. In contrast, 3DP scaffolds did not show significant size changes, indicating that they were more conducive to mechanical stability.

Since mineralization could enhance mechanical properties, scaffolds were subjected to the DMA compression test before and after implantation to evaluate their mechanical enhancement. As shown in Figure 5C, HGel‐*g*‐nHAp had a significantly higher storage modulus than HGel‐µHAp before implantation, indicating that chemically integrating nHAp enhanced the mechanical properties, which was consistent with the results of Figure 2A–E. All three scaffold groups showed different mineralization degrees. HGel‐*g*‐nHAp presented the highest mineralization performance with a 25.3‐fold increase in storage modulus in comparison to preimplantation, while HGel‐µHAp increased only 7.9 times. Micro‐computed tomography (CT) 3D reconstruction (Figure 5D) showed that the greatest number of minerals were deposited on the surface of HGel‐*g*‐nHAp scaffold, while SEM and energy dispersive X‐ray spectroscopy (EDS) mapping (Figure 5D) also confirmed the presence of more uniform calcium/phosphorus fibers on the HGel‐*g*‐nHAp scaffold. Semiquantitative analysis of bone mineral density (BMD) and bone volume fraction (BV/TV) supported the mineralization trend (HGel‐*g*‐nHAp > HGel‐µHAp > hydrogel; *p* < 0.01; Figure 5E). Cell morphology on scaffolds observed by confocal laser scanning microscopy (CLSM) showed that nuclei numbers were significantly higher in the HGel‐*g*‐nHAp group, which also had the largest f‐actin spreading area (Figure 5D). Cell numbers were also confirmed by semiquantitative Image J statistics (*p* < 0.05; Figure 5E). These results suggested that HGel‐*g*‐nHAp provides a more favorable microenvironment for BMSCs adhesion and spreading in the subcutaneous surroundings of nude mice.

The expression levels of osteogenesis‐related (*Runx2* and *OPN*) and angiogenesis‐related (*Vegfa*) genes were examined by qPCR (Figure 5F). The HGel‐*g*‐nHAp and HGel‐µHAp groups had significantly higher expression levels than the hydrogel group, especially *OPN* in the HGel‐*g*‐nHAp group (*p* < 0.05). IHC staining and semiquantitative statistics are shown in Figure 5G,H. The highest positive expression indicated that HGel‐*g*‐nHAp exhibited the greatest ectopic osteogenesis capacity.

### 2.6 HGel‐*g*‐nHAp Accelerates Bone Regeneration in Rabbit Critical‐Size Cranial Defects

**Figure 6:**
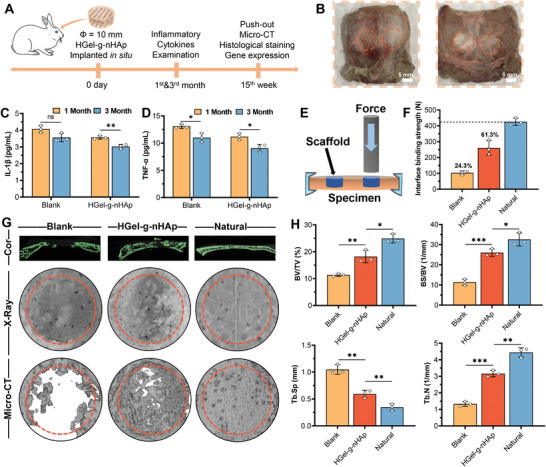
Bone reconstruction in rabbit cranial defect. A) Schematic diagram of in vivo bone repair experiments (*n* = 3). B) Gross view of cranial defects repair after 15 weeks. C,D) Expression of inflammatory cytokines 1L‐1*β* and TNF‐*α* at first and third month. E) Schematic of mechanical push‐out test. F) Interface binding strength between new bone and host tissue of different groups. G) Representative coronal view, X‐ray images, and micro‐CT 3D reconstruction of different treated groups after 15 weeks of implantation. H) Semiquantitative results of bone morphology parameters. Significance level was set as *p* < 0.05 (*), *p* < 0.01 (**), and *p* < 0.001 (***).

Cell‐free HGel‐*g*‐nHAp scaffolds were implanted in situ (rabbit cranial critical‐size defect, *Φ* = 10 mm) and examined for long‐term bone regeneration capacity (**Figure** **6**A and Figure S8, Supporting Information). Figure 6B shows the gross view of defect repair at 15 weeks. The implanted HGel‐*g*‐nHAp scaffold effectively filled the defect area. It formed an integrated fusion with the surrounding host tissue, and the scaffold surface, pores, and edges were replaced by new bone tissue. Inflammatory cytokine (interleukin [IL]‐1*β* and tumor necrosis factor alpha [TNF‐*α*]) levels were lower in the HGel‐*g*‐nHAp group than in the blank group (Figure 6C,D). They significantly downregulated after 3 months, indicating that the HGel‐*g*‐nHAp scaffold had good biosafety and compatibility, and did not cause significant inflammatory reactions. In addition, the HGel‐*g*‐nHAp scaffold showed early proinflammatory and late anti‐inflammatory features, which positively influenced the regulation of bone regeneration.

Interface binding strength between new bone and host tissue was assessed by a push‐out experiment as illustrated in Figure 6E. The fracture load between the HGel‐*g*‐nHAp scaffold and host tissue was 260.7 ± 37.6 N (reaching 61.3% of natural bone), significantly higher than in the blank group (*p* < 0.05; Figure 6F). Micro‐CT and X‐ray were used to visualize the effect of HGel‐*g*‐nHAp scaffold in promoting bone reconstruction (Figure 6G). Compared with the blank group, which showed limited bone formation only at the margins, the HGel‐*g*‐nHAp scaffold accelerated new bone growth with bone healing degrees close to natural bone. A CT semiquantitative analysis (Figure 6H) showed that the HGel‐*g*‐nHAp scaffold had significantly higher BV/TV (18.2 ± 1.9%; 73.1% of natural bone), BS/BV (26.03 ± 1.47 mm^−1^), and trabecular number (Tb.N at 3.1548 ± 0.1566 mm^−1^) but significantly lower trabecular separation (Tb.Sp at 0.5933 ± 0.0544 mm) than the blank group, which were much closer to the natural group. These results suggested that the HGel‐*g*‐nHAp scaffold has a promising bone regeneration performance in rabbit cranial critical‐size defects.

**Figure 7:**
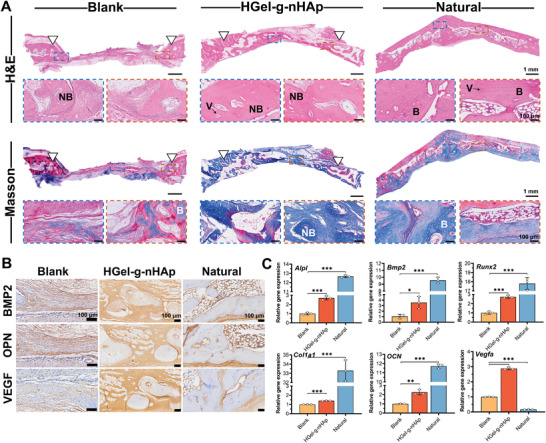
Histological evaluation and genes expression after implantation. A) H&E staining images and Masson's trichrome staining images of different treated groups after 15 weeks of implantation. (B: bone tissue. NB: new bone tissue. V: new blood vessels). Scale bar: 1 mm, 100 µm. B) Representative IHC staining images of BMP2, OPN, and VEGF of different treated groups after 15 weeks of implantation. Scale bar: 100 µm. C) Relative expression levels of osteogenesis‐related genes (*Alpl*, *Bmp2*, *Runx2*, *Col1a1*, *OCN*) and angiogenesis‐related genes (*Vegfa*). Significance level was set as *p* < 0.05 (*), *p* < 0.01 (**), and *p* < 0.001 (***).

Furthermore, defective area tissues were sectioned and stained. H&E and Masson's trichrome staining (**Figure** **7**A) showed neovascularization and mature new bone tissue regeneration in the HGel‐*g*‐nHAp group in comparison to the blank group, which more closely resembled the natural group. IHC staining (Figure 7B) indicated that the ECM of HGel‐*g*‐nHAp group presented higher positive expression of BMP2, OPN, and VEGF than the blank group. In addition, the expression levels of osteogenesis‐related (*Alpl, Bmp2, Runx2*, *Col1a1, OCN*) and angiogenesis‐related (*Vegfa*) genes were significantly higher in the HGel‐*g*‐nHAp group than in the blank group, confirming that the HGel‐*g*‐nHAp scaffold could genetically promote vascularization and bone regeneration in situ.

## 3 Discussion

Natural bone derives its mechanical properties from a highly integrated nanoscale architecture composed of organic (mainly collagen) and inorganic (mainly nHAp) components.^[^ ^18^ ^]^ Researchers have endeavored to enhance the mechanical properties of artificial bone implants by optimizing the interfaces between organic and inorganic phases to mimic natural bone's nanocomposite structure.^[^ ^34^, ^42^, ^43^, ^44^ ^]^ Here, nHAp was endowed with vinyl by silicon‐based coupling agent and coupled with GelMA by photoinitiation to prepare a chemically integrated 3DP scaffold with gelatin grafting nHAp (Gel‐*g*‐nHAp; Figures 1 and 2). Nanostructured optimization improved the mechanical properties, including elasticity, tensile strength, and compressive strength (Figure 2A–E) in comparison to the conventional Gelatin/µHAp composite scaffold, which was beneficial in maintaining scaffold stability to promote new bone formation.

Osteoblasts produce and mineralize new bone matrix during bone regeneration to repair bone defects.^[^ ^45^ ^]^ While some progress has been made by adding exogenous stem cells or growth factors to BTE scaffolds,^[^ ^46^, ^47^ ^]^ their unpredictable clinical risks and cumbersome regulatory approvals have limited their practical application, making cell‐free scaffolds important for regenerating bone defects.^[^ ^4^ ^]^ With the increasing development of bone tissue engineering, the chemical modification of nanomaterial scaffolds could confer satisfactory biological functions by enhancing their specific chemical characteristics.^[^ ^48^, ^49^ ^]^ Catechol‐functionalized hydrogels have been reported to facilitate cell adhesion by bonding particular functional groups (e.g., amine and thiol) in proteins.^[^ ^50^ ^]^ Therefore, the 3DP Gel‐*g*‐nHAp scaffold was further chemically anchored by bioactive hydrogel matrix composed of HADA and Col I to fabricate a biologically functionalized nanostructured scaffold (HGel‐*g*‐nHAp; Figure 2F). This approach avoided excessive scaffold degradation (Figure 2H–J) and promoted BMSCs proliferation, adhesion, and osteogenic differentiation (Figure 3) by constructing a fibrillar interwoven osteogenic microenvironment mimicking the ECM.

This study confirmed that the cell‐free HGel‐*g*‐nHAp scaffold could stimulate rapid vascularization and early osteogenesis by recruiting ESCs in situ (Figure 4). This specific biofunction might be attributed to the following three aspects: first, nanostructured scaffolds provide architectural support to maintain the transport space of cell/functional factor, while the alterations of nanotopography might affect the mechano‐sensitivity of stem cells and regulate their fate, including proliferation, adhesion, and osteogenic differentiation.^[^ ^51^ ^]^ Second, the silicon element was introduced into the scaffold through silicon‐based coupling agent, which might promote early angiogenesis and osteogenesis.^[^ ^39^, ^41^ ^]^ Finally, our newly reported research had proved that dopamine‐integrated nanointerface between fibrillar matrix and nHAp could drove functional cells/cytokine adhesion, inchoate vascularization, and greatly enhance endogenous stem cell recruitment to initiate robust osteogenesis.^[^ ^52^ ^]^ Hence, the photointegrated interface binding and dopamine‐mediated multiple chemical reactions in HGel‐g‐nHAp might contribute to ESCs recruitment and provide a biomimetic niche for stem cells to guide early osteogenesis and stimulate rapid vascularization.

Next, the BMSCs‐laden HGel‐*g*‐nHAp scaffold showed enhanced mineral deposition in an ectopic osteogenesis nude mouse model (Figure 5A–D). For unnanostructured Gel‐µHAp 3DP scaffold, although similarly anchored by dopamine‐functionalized hydrogels, it showed lower ectopic osteogenic capacity than the chemically integrated scaffold subcutaneously in nude mice (hydrogel < HGel‐µHAp < HGel‐*g*‐nHAp; Figure 5). Among the three groups, the nanostructured scaffold showed the highest ectopic mineralization capacity, with significantly upregulated expression and secretion of osteogenesis‐related genes and matrix (Figure 5E–H), confirming the osteogenic advantages of chemically integrated nanostructured scaffolds. After being implanted into a rabbit cranial critical‐size defect for 15 weeks, the HGel‐*g*‐nHAp scaffold achieved considerable osseointegration and new bone regeneration by increasing the expression of relevant genes and specific matrix (Figures 6 and 7). These results implied the potential of photointegrated nanostructured 3D‐printed scaffolds for bone regeneration.

## 4 Conclusion

In summary, we have developed a chemically integrated nanostructured 3DP scaffold by photointegrating nHAp and gelatin via carbon–carbon double bonding. A dopamine‐functionalized hydrogel was further anchored to impart specific biological functions. The nanostructured 3DP hybrid scaffold greatly improved its mechanical properties, accelerated BMSCs proliferation and osteogenic differentiation in vitro, and stimulated ESCs recruitment and early osteogenesis in vivo. Benefiting from nanostructures resembling natural bone, the scaffold enabled substantial ectopic osteogenesis and in situ bone reconstruction. This methodology might present a new strategy for developing nanobiomaterials for bone regeneration.

## 5 Experimental Section

### Materials

Nanohydroxyapatite (HAp) slurry, µHAp spherical particles (size of 20–50 µm), and Collagen type I (extracted from calfskin) were obtained from National Engineering Research Center for Biomaterials, Sichuan University, Chengdu, China. TOES, YDH‐151, and lithium phenyl(2,4,6‐trimethylbenzoyl) phosphinate (LAP) were purchased from Shanghai Aladdin Biochemical Technology Co., Ltd., China. Ethanol, acetic acid, and sodium carboxymethyl cellulose were purchased from Chengdu Kelong Chemical Technology Co., Ltd., China. HADA and GelMA were synthesized following the previous report.^[^ ^6^, ^7^ ^]^ *α*‐Minimum essential medium (*α*‐MEM), fetal bovine serum (FBS), and penicillin/streptomycin (PS) were obtained from Hyclone, USA. Fluorescein diacetate (FDA), propidium iodide (PI), Triton X‐100, rhodamine‐phalloidin, and 4′,6‐diamidino‐2‐phenylindole (DAPI) were purchased from Sigma‐Aldrich, USA.

### Preparation and Characterization of Vinyl‐Modified Nanohydroxyapatite

Vinyl‐modified nanohydroxyapatite (vinyl‐nHAp) was synthesized based on that previously reported with minor modifications.^[^ ^34^ ^]^ Briefly, 5 mL TEOS and YDH‐151 were, respectively, added dropwise into a 100 mL aqueous ethanol solution (pH 4.0, ethanol/water = 6–9) and stirred for 2 h to hydrolyze silane. Then nanohydroxyapatite slurry containing 5 g nHAp was added into the above solution and stirred for 12 h. The reaction mixture was adjusted to pH 8.0 using 10% NaOH and the product was washed several times with ethanol, water and dried. The chemical structures of nHAp and four groups of vinyl‐nHAp were measured via FT‐IR (Bruker, Vertex 70, Germany). Iodometry was used to determine the concentration of vinyl. The solid content of hydroxyapatite was measured by a thermogravimetric analyzer (TA, Q500, USA) in the atmosphere of nitrogen. XRD (PANalytical, Netherlands) and XPS (Kratos, UK) were used to determine crystal phase identification and elemental analysis of vinyl‐nHAp. HAp particle size and agglomeration were measured by DLS (Zetasizer Nano, Malvern, UK) with water as a medium. The micro‐ and nanostructure were observed by SEM (Hitachi, S‐4800, Japan) and TEM (JEOL, JEM‐2100Plus, Japan). Image J was used to semiquantified nHAp granularity according to the TEM images.

### Fabrication and Characterization of Gel‐g‐nHAp 3DP Scaffold

To prepare 3D‐printed bioink, µHAp/vinyl‐nHAp (3.75 g), sodium carboxymethyl cellulose (37.5 mg), and photoinitiator LAP (20 mg) were added to 5 mL of 15% GelMA solution. The above materials were put into a homogenizer (ARE‐310, THINKY, Japan), mixed, and degassed thoroughly. To create mechanical test splines, various bioinks were poured into different molds and crosslinked using blue light. Dynamic mechanical analysis (DMA, TA, Q800, USA) test conditions were set as follows: a prestress force of 5 mN, an amplitude of 20 µm, and a frequency of 20 Hz (compress); a prestress force of 1 mN, a stretching speed of 2 mm min^−1^ (stretch). Compressive strength of spline (*d* = 6 mm, *h* = 8 mm) was measured by a universal testing machine (Shimadzu, AGS‐X, Japan) using a speed of 2 mm min^−1^.

To prepare 3DP scaffolds, bioinks were transferred into the printing needle (*d* = 0.4 mm) and printed by a direct ink writing printer (Bio‐Architect‐Pro, Regenovo Biotech. Co. Ltd., China) using the following conditions: an extrusion pressure of 0.3 MPa, a printing speed of 5–10 mm s^−1^, and a nozzle temperature of 32 °C. The printed scaffold was cured by blue light (*λ* = 405 nm) irradiation. To prepare bioactive hydrogels, HADA and Col I were dissolved in 0.1 m acetic acid in 10 mg mL^−1^ concentrations. Gel‐*g*‐nHAp 3DP scaffolds were immersed in the above pre‐gel solution, crosslinked by raising the pH to 7.5, and freeze‐dried for 12 h. The fiber and pore sizes of scaffolds were semiquantified by Image J software. Weight and size change ratios were used to depict the swelling of scaffolds in PBS. Tris‐HCl buffer was used to characterize the degradation behavior of scaffolds and the volume of buffer was about 200 mL g^−1^ to the mass of scaffold.

### Cell Proliferation and Differentiation In Vitro

BMSCs were extracted from newborn rabbit bone marrow and expanded to Passage 3. To establish a materials‐cells cocultured complex, 20 µL of 5 × 10^6^ mL^−1^ BMSC suspension was added to the scaffold surface and penetrated. The complex was cultured in *α*‐MEM containing 10% FBS and 1% PS in an ultralow attachment 24‐well plate (Costar, USA). Each well contained 2 mL medium and the medium was rechanged every 2 days. To evaluate the biocompatibility of scaffolds, CCK‐8 test was performed after different days of culturing. Briefly, the complex was incubated in 10% CCK‐8 solution for 2 h, and cell density was evaluated by measuring the OD value at 450 nm. Additionally, live/dead staining of BMSCs was observed via CLSM (LSM 880, ZEISS) by staining cells with 1 µg mL^−1^ of FDA and PI. After fixing with 4% paraformaldehyde for 30 min and transparentizing with 0.1% Triton X‐100 for 5 min, the scaffolds were stained with rhodamine‐phalloidin (5 µg mL^−1^) for 6 h and DAPI (10 µg mL^−1^) for 2 min. The complex was immobilized by 2.5% glutaraldehyde, dehydrated by gradient ethanol, and sprayed with gold. Cytoskeleton and spreading morphology of BMSCs on the scaffolds were observed by CLSM and SEM.

Real‐time quantitative PCR was used to detect the relative expression levels of osteogenesis‐related genes after different days of coculturing. Total RNA of BMSCs on the scaffolds was extracted by RNeasy Mini Kit (Qiagen) and reversed to cDNA using iScript cDNA Synthesis Kit (Bio‐Rad). The yield of RNA was determined using a NanoDrop 2000 spectrophotometer (Thermo Scientific, USA). Quantitative RT‐PCR was performed using SYBR Green Supermix (Roche, USA) on CFX96 RT‐PCR detection system (Bio‐Rad). *GAPDH* was used as an internal reference and relative gene expression levels were calculated by the 2^−ΔΔCt^ method. The primers used are listed in Table S1 in the Supporting Information. After fixing with 4% paraformaldehyde, the scaffolds were paraffin‐embedded and sectioned. H&E, ARS, Masson's trichrome staining, and IHC staining were carried out to further evaluate osteogenic and angiogenic differentiation of BMSCs. For IHC staining, slices were treated in citric acid antigen retrieval solution for 1 h before being incubated with primary antibody at 37 °C for 1 h, then washed three times with PBS, and treated for another hour with the secondary antibody.

### Endogenous Stem Cells Recruitment and Early Osteogenesis In Vivo

A rabbit skull defect model (Φ = 10 mm) was used to evaluate the recruitment ability of endogenous stem cells. All animal studies were approved by the Sichuan University Medical Ethics Committee (KS2022903). All animal procedures were performed in accordance with the Guidelines for Care and Use of Laboratory Animals of Sichuan University. Gel‐*g*‐nHAp and HGel‐*g*‐nHAp scaffolds were implanted in situ and the implants were taken out on different days for H&E, IHC staining, immunofluorescence (IF) staining, and RT‐PCR. Mean fluorescence intensity of IF staining was semiquantified by Image J software.

### Ectopic Osteogenesis in Subcutaneous Implantation Model

The ectopic mineral deposition of scaffolds was evaluated using a subcutaneous implantation model in nude mice. BMSCs were seeded onto the surface of scaffolds (*d* = 8 mm, *h* = 2 mm) at a concentration of 2 × 105 cells per scaffold. An intraperitoneal dose of pentobarbital sodium was used to anesthetize nude mice before implanting BMSCs‐laden scaffolds subcutaneously. After 30 days, the nude mice were euthanized, and the diameters of the explants were measured. The storage modulus of scaffolds was measured by DMA before and after implantation. Micro‐CT (VivaCT80, Scanco Medical AG) was utilized to visualize the effect of mineral deposition and quantify BV/TV and BMD at 15 µm volume pixel. Mimics software (Materialize) was used to reconstruct the 3D image of mineralized scaffolds. The scaffolds were stained with rhodamine‐phalloidin/DAPI and observed by CLSM and the cell number was semiquantified by Image J. After dehydration, the scaffolds were observed by SEM to evaluate mineralized hydroxyapatite deposition and calcium/phosphorus element mapping was employed to locate mineral deposition. RT‐PCR, H&E, and IHC staining (RUNX2, OCN, and VEGF) were conducted as mentioned above. Image J was used to calculate semiquantitative results of average optical density (AOD value) for IHC staining.

### Bone Reconstruction in Rabbit Critical‐Sized Cranial Defect Model

A critical‐size defect (Φ = 10 mm) model was constructed in the skull of New Zealand Rabbits (male, 2.5–3 kg, 2.5–3 months old) to evaluate the bone regeneration potential of scaffolds (*n* = 12). After an appropriate dosage of sodium pentobarbital was administered intravenously to the rabbits, the skin was shaved and disinfected with iodophor, followed by an incision of the skin and periosteum to expose the skull. A trephine machine was used to create two critical‐size defects of 10 mm diameter, and saline was supplied dropwise to reduce the temperature during drilling. The wounds were carefully sutured after scaffolds implantation in the defects and disinfected with iodophor, followed by injection of 2 mL of gentamicin sulfate. Untreated cranial defects were set as Blank group, while rabbits without any surgery were set as Natural group. Whole blood was collected 1 month and 3 months after implantation, and the concentrations of inflammatory cytokines TNF‐*α* and IL‐1*β* were determined using enzyme‐linked immunosorbent assay kits. Rabbits were euthanized after 15 weeks post‐surgery, and the implanted scaffolds with surrounding cranial bone tissue were removed. Samples were stored in liquid nitrogen to extract total RNA for osteogenic and angiogenic gene expression analyses. Paraformaldehyde fixation was used for other assays. A push‐out model was used to examine the scaffold's integration with the surrounding skull, and the samples at the defective site were pushed out using mechanical testing machine (Shimadzu, EZ‐LX 1 kN, Japan). Micro‐CT (VivaCT80, Scanco Medical AG) was utilized to visualize cranial bone regeneration, and BV/TV, bone surface/bone volume (BS/BV), number of bone trabeculae (Tb.N), and bone trabeculae separation (Tb.Sp) were semiquantified. Samples were decalcified in 10% ethylenediaminetetraacetic acid solution for 2 months, then dehydrated in gradient alcohol, paraffin‐embedded, and sectioned for H&E, Masson's trichrome, and IHC staining.

### Statistical Analysis

All the data in this study were presented as the mean value ± standard deviation (SD) of more than three independent experiments. All the data and statistically differences between groups were analyzed using GraphPad Prism software (GraphPad Software Inc.) by Student's *t*‐test, one‐way analysis of variance, and followed by Tukey's post hoc test. Significance level was set as *p* < 0.05 (*), *p* < 0.01 (**), and *p* < 0.001 (***).

## Conflict of Interest

The authors declare no conflict of interest.

## Supporting information

Supporting Information

## Data Availability

The data that support the findings of this study are available from the corresponding author upon reasonable request.

## References

[1] W. H. Wang , K. W. K. Yeung , Bioact. Mater. 2017, 2, 224. 29744432 10.1016/j.bioactmat.2017.05.007 PMC5935655

[2] C. Q. Zhang , D. A. Mcadams , J. C. Grunlan , Adv. Mater. 2016, 28, 6292. 27144950 10.1002/adma.201505555

[3] J. A. Burdick , R. L. Mauck , J. H. Gorman , R. C. Gorman , Sci. Transl. Med. 2013, 5, 176ps4. 10.1126/scitranslmed.3003997 PMC4041107 23486777

[4] A. K. Gaharwar , I. Singh , A. Khademhosseini , Nat. Rev. Mater. 2020, 5, 686.

[5] J. R. Xavier , T. Thakur , P. Desai , M. K. Jaiswal , N. Sears , E. Cosgriff‐Hernandez , R. Kaunas , A. K. Gaharwar , ACS Nano 2015, 9, 3109. 25674809 10.1021/nn507488s

[6] G. Lu , Y. Xu , Q. Liu , M. Chen , H. Sun , P. Wang , X. Li , Y. Wang , X. Li , X. Hui , E. Luo , J. Liu , Q. Jiang , J. Liang , Y. Fan , Y. Sun , X. Zhang , Nat. Commun. 2022, 13, 2499. 35523800 10.1038/s41467-022-30243-5 PMC9076642

[7] X. Pu , L. Tong , X. Wang , Q. Liu , M. Chen , X. Li , G. Lu , W. Lan , Q. Li , J. Liang , Y. Sun , Y. Fan , X. Zhang , ACS Appl. Mater. Interfaces 2022, 14, 20591. 35500105 10.1021/acsami.1c25015

[8] L. Li , J. Li , J. Guo , H. Zhang , X. Zhang , C. Yin , L. Wang , Y. Zhu , Q. Yao , Adv. Funct. Mater. 2019, 29, 1807356.

[9] J. O. Akindoyo , M. D. H. Beg , S. Ghazali , H. P. Heim , M. Feldmann , Composites, Part A 2017, 103, 96.

[10] A. Faingold , S. R. Cohen , N. Reznikov , H. D. Wagner , Acta Biomater. 2013, 9, 5956. 23220032 10.1016/j.actbio.2012.11.032

[11] H. Ping , W. Wagermaier , N. Horbelt , E. Scoppola , C. Li , P. Werner , Z. Fu , P. Fratzl , Science 2022, 376, 188. 35389802 10.1126/science.abm2664

[12] G. L. Koons , M. Diba , A. G. Mikos , Nat. Rev. Mater. 2020, 5, 584.

[13] U. G. K. Wegst , H. Bai , E. Saiz , A. P. Tomsia , R. O. Ritchie , Nat. Mater. 2015, 14, 23. 25344782 10.1038/nmat4089

[14] L. Zhu , D. Luo , Y. Liu , Int. J. Oral Sci. 2020, 12, 6. 32024822 10.1038/s41368-020-0073-y PMC7002518

[15] H. Zhu , M. Monavari , K. Zheng , T. Distler , L. Ouyang , S. Heid , Z. Jin , J. He , D. Li , A. R. Boccaccini , Small 2022, 18, 2104996. 10.1002/smll.202104996 35102718

[16] G. Cheng , C. Yin , H. Tu , S. Jiang , Q. Wang , X. Zhou , X. Xing , C. Xie , X. Shi , Y. Du , H. Deng , Z. Li , ACS Nano 2019, 13, 6372. 31184474 10.1021/acsnano.8b06032

[17] H. Li , D. He , X. Xiao , G. Yu , G. Hu , W. Zhang , X. Wen , Y. Lin , X. Li , H. Lin , Y. Diao , Y. Tang , ACS Appl. Mater. Interfaces 2021, 13, 25290. 33908252 10.1021/acsami.1c05437

[18] T. Gong , J. Xie , J. F. Liao , T. Zhang , S. Y. Lin , Y. F. Lin , Bone Res. 2015, 3, 15029. 26558141 10.1038/boneres.2015.29 PMC4639780

[19] M. N. Collins , G. Ren , K. Young , S. Pina , R. L. Reis , J. M. Oliveira , Adv. Funct. Mater. 2021, 31, 2010609.

[20] F. P. W. Melchels , M. A. N. Domingos , T. J. Klein , J. Malda , P. J. Bartolo , D. W. Hutmacher , Prog. Polym. Sci. 2012, 37, 1079.

[21] P. Gu , Y. Xu , Q. Liu , Y. Wang , Z. Li , M. Chen , R. Mao , J. Liang , X. Zhang , Y. Fan , Y. Sun , ACS Appl. Mater. Interfaces 2022, 14, 32914. 10.1021/acsami.2c07649 35829709

[22] V. Fitzpatrick , Z. Martín‐Moldes , A. Deck , R. Torres‐Sanchez , A. Valat , D. Cairns , C. Li , D. L. Kaplan , Biomaterials 2021, 276, 120995. 34256231 10.1016/j.biomaterials.2021.120995 PMC8408341

[23] A. G. Kurian , R. K. Singh , K. D. Patel , J. H. Lee , H. W. Kim , Bioact. Mater. 2022, 8, 267. 34541401 10.1016/j.bioactmat.2021.06.027 PMC8424393

[24] F. Gao , Z. Xu , Q. Liang , H. Li , L. Peng , M. Wu , X. Zhao , X. Cui , C. Ruan , W. Liu , Adv. Sci. 2019, 6, 1900867. 10.1002/advs.201900867 PMC6685475 31406678

[25] J. Gao , X. Ding , X. Yu , X. Chen , X. Zhang , S. Cui , J. Shi , J. Chen , L. Yu , S. Chen , J. Ding , Adv. Healthcare Mater. 2021, 10, 2001404. 10.1002/adhm.202001404 33225617

[26] B. H. Atak , B. Buyuk , M. Huysal , S. Isik , M. Senel , W. Metzger , G. Cetin , Carbohydr. Polym. 2017, 164, 200. 28325318 10.1016/j.carbpol.2017.01.100

[27] C. Shao , R. Zhao , S. Jiang , S. Yao , Z. Wu , B. Jin , Y. Yang , H. Pan , R. Tang , Adv. Mater. 2018, 30, 1704876. 10.1002/adma.201704876 29315839

[28] M. Shen , C. Wang , D. Hao , J. Hao , Y. Zhu , X. Han , L. Tonggu , J. Chen , K. Jiao , F. R. Tay , L. Niu , Adv. Mater. 2022, 34, 2107924. 10.1002/adma.202107924 34850469

[29] H. S. Kim , J. H. Lee , N. Mandakhbayar , G. Z. Jin , S. J. Kim , J. Y. Yoon , S. B. Jo , J. H. Park , R. K. Singh , J. H. Jang , U. S. Shin , J. C. Knowles , H. W. Kim , Biomaterials 2021, 274, 120857. 33965799 10.1016/j.biomaterials.2021.120857

[30] P. Song , M. Li , B. Zhang , X. Gui , Y. Han , L. Wang , W. Zhou , L. Guo , Z. Zhang , Z. Li , C. Zhou , Y. Fan , X. Zhang , Composites, Part B 2022, 244, 110163.

[31] X. Xue , Y. Hu , S. Wang , X. Chen , Y. Jiang , J. Su , Bioact. Mater. 2022, 12, 327. 35128180 10.1016/j.bioactmat.2021.10.029 PMC8784310

[32] Y. Yang , T. Xu , Q. Zhang , Y. Piao , H. P. Bei , X. Zhao , Small 2021, 17, 2006598. 10.1002/smll.202006598 33705605

[33] J. Tang , K. Xi , H. Chen , L. Wang , D. Li , Y. Xu , T. Xin , L. Wu , Y. Zhou , J. Bian , Z. Cai , H. Yang , L. Deng , Y. Gu , W. Cui , L. Chen , Adv. Funct. Mater. 2021, 31, 2102465.

[34] X. Li , M. Y. Chen , P. L. Wang , Y. Yao , X. W. Han , J. Liang , Q. Jiang , Y. Sun , Y. J. Fan , X. D. Zhang , Nanoscale 2020, 12, 12869. 32520065 10.1039/d0nr01824d

[35] C. S. Goonasekera , K. S. Jack , J. J. Cooper‐White , L. Grøndahl , J. Mater. Chem. B 2013, 1, 5842. 32261241 10.1039/c3tb21110j

[36] O. Bareiro , L. A. Santos , Colloids Surf., B 2014, 115, 400. 10.1016/j.colsurfb.2013.12.027 24503294

[37] C. Shuai , L. Yu , P. Feng , C. Gao , S. Peng , Colloids Surf., B 2020, 193, 111083. 10.1016/j.colsurfb.2020.111083 32388393

[38] V. Bunpetch , X. Zhang , T. Li , J. Lin , E. P. Maswikiti , Y. Wu , D. Cai , J. Li , S. Zhang , C. Wu , H. Ouyang , Biomaterials 2019, 192, 323. 30468999 10.1016/j.biomaterials.2018.11.025

[39] H. Li , K. Xue , N. Kong , K. Liu , J. Chang , Biomaterials 2014, 35, 3803. 24486216 10.1016/j.biomaterials.2014.01.039

[40] H. Zhang , C. Qin , M. Zhang , Y. Han , J. Ma , J. Wu , Q. Yao , C. Wu , Nano Today 2022, 46, 101584.

[41] X. Wang , L. Gao , Y. Han , M. Xing , C. Zhao , J. Peng , J. Chang , Adv. Sci. 2018, 5, 1800776. 10.1002/advs.201800776 PMC6247030 30479923

[42] X. D. Hou , Y. X. Chen , F. Chen , J. F. Liu , T. L. Wang , Y. P. Luo , S. H. Jia , P. Wang , S. Tan , B. Q. Lu , Z. F. Zhou , L. P. Zheng , Composites, Part B 2022, 228, 109396.

[43] D. H. Liu , W. Nie , D. J. Li , W. Z. Wang , L. X. Zheng , J. T. Zhang , J. L. Zhang , C. Peng , X. M. Mo , C. L. He , Chem. Eng. J. 2019, 362, 269.

[44] Z. Hussain , I. Ullah , X. Liu , W. Shen , P. Ding , Y. Zhang , T. Gao , M. Mansoorianfar , T. Gao , R. Pei , Chem. Eng. J. 2022, 438, 135611.

[45] A. Wubneh , E. K. Tsekoura , C. Ayranci , H. Uludağ , Acta Biomater. 2018, 80, 1. 30248515 10.1016/j.actbio.2018.09.031

[46] T. N. Vo , F. K. Kasper , A. G. Mikos , Adv. Drug Delivery Rev. 2012, 64, 1292. 10.1016/j.addr.2012.01.016 PMC3358582 22342771

[47] J. Tan , M. Zhang , Z. Hai , C. Wu , J. Lin , W. Kuang , H. Tang , Y. Huang , X. Chen , G. Liang , ACS Nano 2019, 13, 5616. 31059238 10.1021/acsnano.9b00788

[48] S. S. Lee , X. Du , I. Kim , S. J. Ferguson , Matter 2022, 5, 2722.

[49] J. Dinoro , M. Maher , S. Talebian , M. Jafarkhani , M. Mehrali , G. Orive , J. Foroughi , M. S. Lord , A. Dolatshahi‐Pirouz , Biomaterials 2019, 214, 119214. 31163358 10.1016/j.biomaterials.2019.05.025

[50] Z. Li , H. Cao , Y. Xu , X. Li , X. W. Han , Y. Fan , Q. Jiang , Y. Sun , X. Zhang , Carbohydr. Polym. 2021, 267, 118224. 34119177 10.1016/j.carbpol.2021.118224

[51] W. L. Murphy , T. C. McDevitt , A. J. Engler , Nat. Mater. 2014, 13, 547. 24845994 10.1038/nmat3937 PMC4163547

[52] X. Li , Z. Li , P. Wang , G. Lu , L. Tong , Q. Liu , Y. Chen , J. Lin , E. Luo , J. Liang , Q. Jiang , Y. Fan , X. Zhang , Y. Sun , Adv. Funct. Mater. 2023, 2212738.

