# Supplementary material for: Nanostructured 3D‐Printed Hybrid Scaffold Accelerates Bone Regeneration by Photointegrating Nanohydroxyapatite
Source: Adv Sci (Weinh). 2023 Mar 11;10(13):2300038. doi: 10.1002/advs.202300038 (PMC10161056; doi:10.1002/advs.202300038)
Supplement: Supplementary file 1 — Supporting Information [file ADVS-10-2300038-s001.pdf]

Supporting Information for

Nanostructured 3D Printed Hybrid Scaffold Accelerates Bone Regeneration by Photo-Integrating Nanohydroxyapatite

Lei Tong, Xiaocong Pu, Quanying Liu, Xing Li, Manyu Chen, Peilei Wang, Yaping Zou, Gonggong Lu, Jie Liang, Yujiang Fan, Xingdong Zhang, and Yong Sun**

E-mail: jie_L88@126.com (J. Liang), sunyong8702@scu.edu.cn (Y. Sun)

This Supporting Information includes:

Figures. S1 to S8

Table. S1

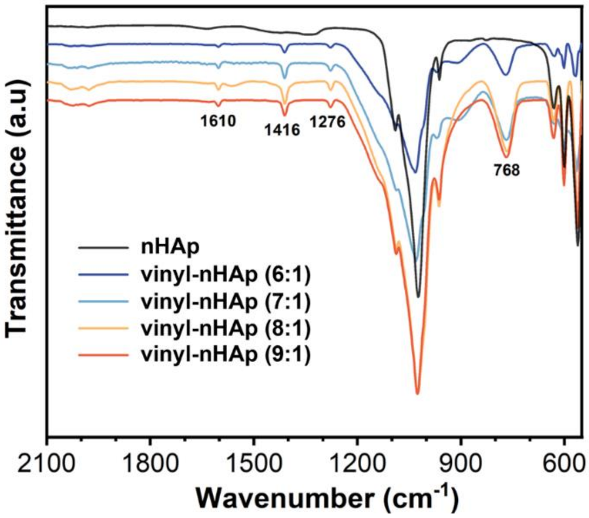

Fig. S1. FT-IR spectra of nHAp and vinyl-nHAps.

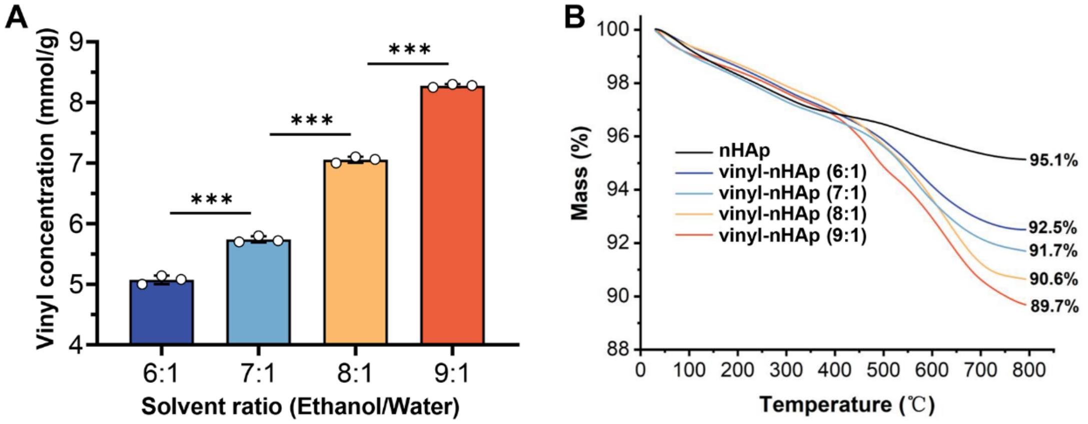

Fig. S2. Vinyl-nHAp has the highest vinyl content when ethanol:water ratio was 9:1. (**A**) Vinyl concentration quantified by iodine titration method. Error bars were presented as standard deviation (SD) of three independent experiments. $p < 0.001$ (***). (**B**) Thermogravimetry curves of nHAp and vinyl-nHAps.

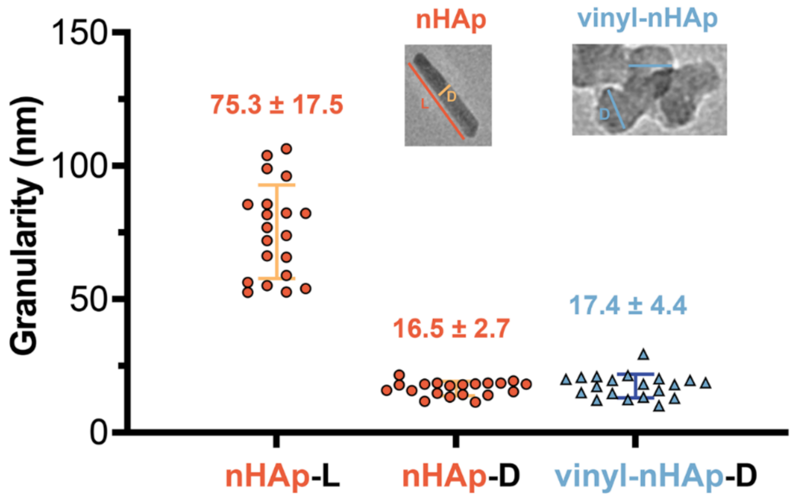

Fig. S3. Semi-quantitative statistics of nHAp/vinyl-nHAp granularity according to TEM images (L: Length, D: Diameter; n = 20).

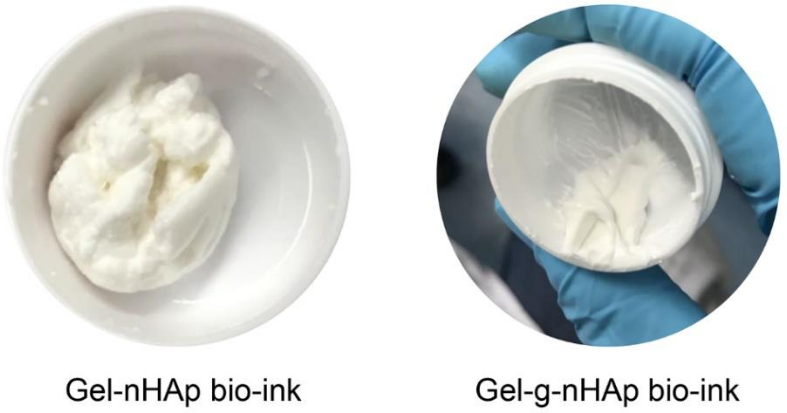

Fig. S4. Gross view images of Gel-nHAp and Gel-g-nHAp bio-inks.

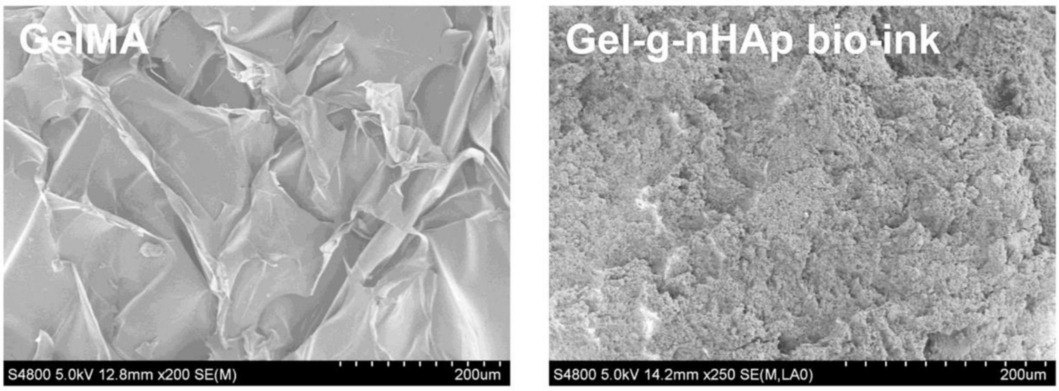

Fig. S5. SEM images of GelMA and Gel-g-nHAp bio-ink.

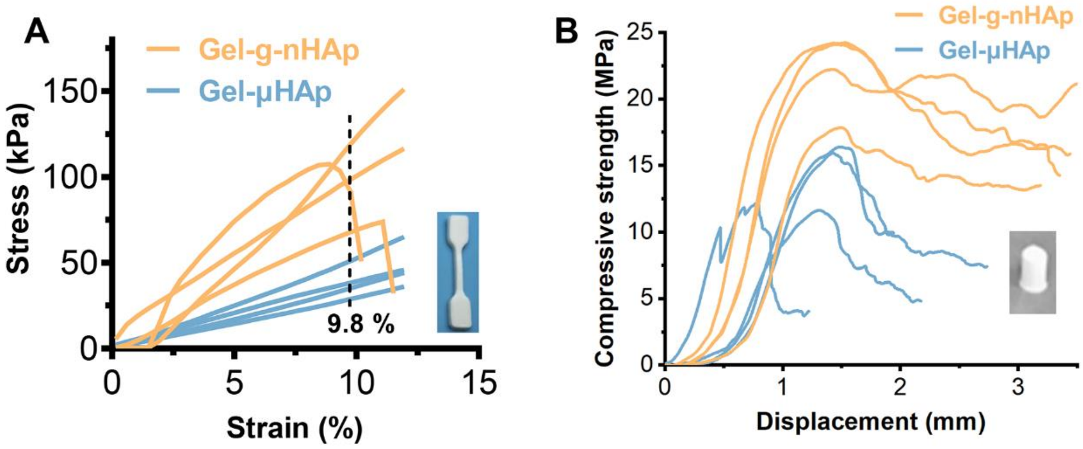

Fig. S6. Gel-g-nHAp showed better mechanical properties than Gel-μHAp. (A) Tensile test measured by DMA. (B) Compressive strength measured by universal testing machine.

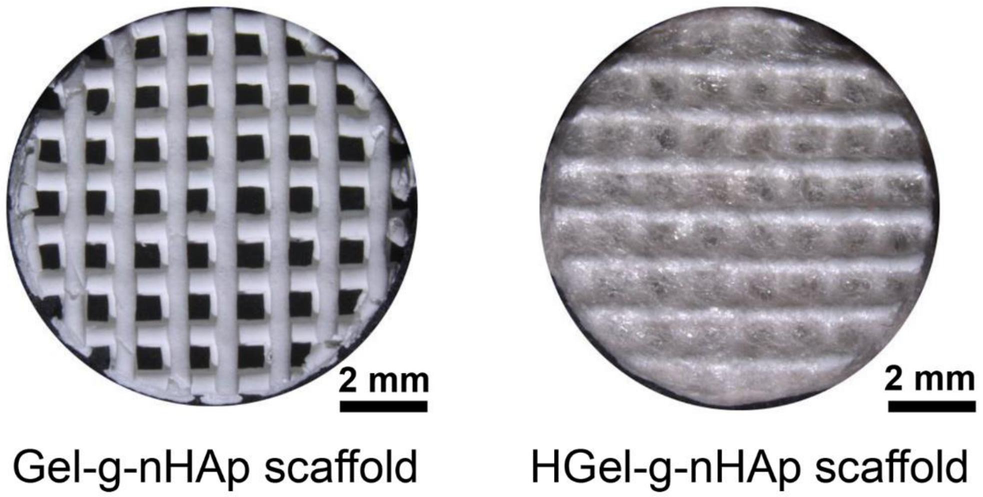

Fig. S7. Gross view images of Gel-g-nHAp and HGel-g-nHAp 3DP scaffolds.

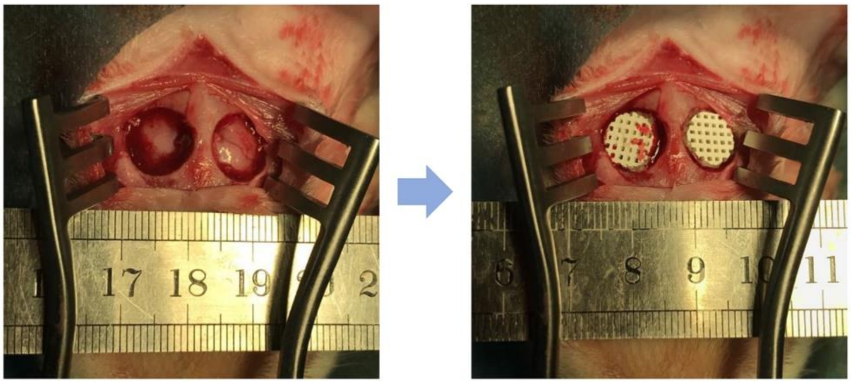

Fig. S8. Surgical procedure of *in situ* skull repair of HGel-g-nHAp 3DP scaffolds in rabbit cranial critical-size defect model.

Table S1. Sequences of primers used for RT-PCR.

Primer names	Sequences (5'-3')
GADPH-F	ACGGATTTGGTCGTATTGGGCG
GADPH-R	CTCCTGGAAGATGGTGATGG
Col1a1-F	CGATGGCTTCCAGTTCGAGT
Col1a1-R	GCTACGCTGTTCTTGCAGTG
Alpl-F	ACCACCACGAGAGTGAACCA
Alpl-R	CGTTGTCTGAGTACCAGTCCC
Bmp2-F	ACAAGTGGGAAAACCACCCG
Bmp2-R	TGATGGAAACCGCTGTCGTC
OCN-F	CAGGCAGAGGCAAAGCCC
OCN-R	AGGGGATCCGGGTAAGGAG
OPN-F	CCGGTTAAACACGCTGAT
OPN-R	CTGCTTCTGAGATGGGTC
Vegfa-F	AGTTCGAGGAAAGGGCAAGG
Vegfa-R	ACGCGAGTCTGTGTTTTTGC
Runx2-F	AGAAGCTTGATGACTCTAAACC
Runx2-R	CTCTCATACTGGGATGAGGAAT
